# A Case of Postpartum Takotsubo (Stress) Cardiomyopathy

**DOI:** 10.1155/2022/4739742

**Published:** 2022-08-04

**Authors:** Fadi Kandah, Tanya Deol, Pooja Dhruva, Zachary Chandler, Thaer Musa, Gladys Velarde

**Affiliations:** ^1^Internal Medicine, UF Health Jacksonville, Dept. of Medicine, 655 W 8th Street, Jacksonville, FL 32209-6595, USA; ^2^Cardiology, UF Health Jacksonville, Dept. of Cardiology, 655 W 8th Street, Jacksonville, FL 32209-6595, USA

## Abstract

Takotsubo (stress) cardiomyopathy (TCM) is usually triggered by psychological and/or physical stress. Most often, it is seen in postmenopausal women. Cases of TCM related to pregnancy are rare. We present a unique case of a 35-year-old, two-day postpartum female who was diagnosed with TCM.

## 1. Introduction

Despite being described in the 1990s, TCM remains poorly understood. While known to be triggered by acute stressors, the exact mechanism is largely unknown. Numerous etiologies have been proposed, including excess catecholamine release during stress, hormones, microvascular ischemia, or spasms [1]. The syndrome mimics an acute coronary syndrome (ACS) presentation with angina-type chest pain, ischemic EKG changes, and elevated cardiac biomarkers. One of the hallmarks of the syndrome is findings on echocardiography which can show severe left ventricular dysfunction with regional dysfunction, most commonly a hypokinetic apex or apical ballooning. EKG abnormalities are very common, including ST elevations and diffuse T wave inversions and prolonged QTc with ST depressions being less common [1, 2]. Additionally, cardiac biomarkers can be elevated, although more modestly elevated compared to ST-elevated myocardial infarctions (STEMI) [2].

## 2. History of Presentation

A 35-year-old African-American woman (gravida 7, para 5) presented for evaluation of sudden onset substernal chest pain, diaphoresis, and dyspnea on exertion. Two days prior to presentation, she had a spontaneous vaginal delivery of a singleton healthy baby at term. Both her pregnancy and delivery were uncomplicated. At presentation, she had a temperature of 98.7°F, blood pressure of 140/87 mmHg, heart rate of 70 beats per minute, respiratory rate of 20 breaths per minute, and oxygen saturation of 97% on room air. Cardiopulmonary examination revealed no accessory muscle use, clear lungs to auscultation bilaterally, a regular rhythm, no cardiac murmurs or rub, and an S3 gallop without jugular venous distension. There was trace peripheral edema, and she had mild suprapubic tenderness with an otherwise unrevealing exam.

## 3. Past Medical History

The patient had a history of Roux-en-Y gastric bypass surgery (four years prior due to morbid obesity) and iron-deficiency anemia due to menorrhagia on oral iron supplementation. She did experience preeclampsia with a prior pregnancy without sequelae. She was a nonsmoker, consumed alcohol socially, and denied any illicit substance use.

## 4. Investigations

**Figure 1:**
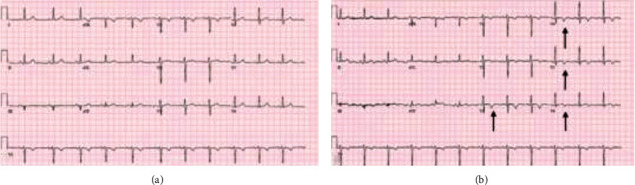
(a) EKG on admission and (b) EKG two hours later showing dynamic T wave changes in leads V3-V6 (demonstrated by the arrows).

Initial chest radiographs and comprehensive metabolic panel were unremarkable. Only abnormality on complete blood count was hemoglobin level of 7.1 g/dL; patient's baseline hemoglobin was 8 g/dL. Chest computed tomography ruled out pulmonary embolism. The initial high-sensitivity troponin T (TnT) was 99 ng/L and 408 ng/L (reference range: 0-14 ng/L) an hour following. The initial electrocardiogram (EKG) showed normal sinus rhythm without acute ischemic findings. EKG performed two hours later showed 0.5 mm ST elevation in leads II and aVF and dynamic T wave inversions in leads III, V3-V6 (Figure 1). The constellation of findings were initially most concerning for spontaneous coronary artery dissection (SCAD).

## 5. Differential Diagnosis

The differential diagnosis in this case included pulmonary emboli, spontaneous coronary artery dissection (SCAD), coronary artery spasm, ischemic obstructive coronary artery disease, peripartum cardiomyopathy, or TCM.

## 6. Management

**Figure 2:**
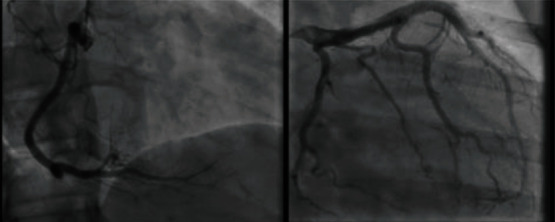
Left heart catheterization demonstrating angiographically normal coronary arteries.

She was initiated on aspirin and heparin infusion and taken urgently for left heart catheterization (LHC). This revealed no evidence of significant coronary artery disease, coronary dissection, spasm, or myocardial bridging (Figure 2).

**Figure 3:**
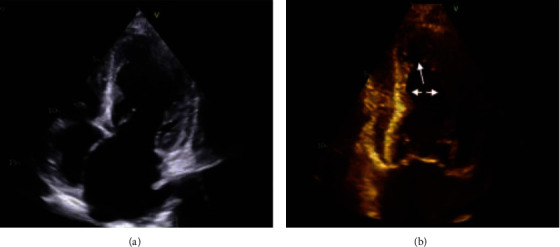
Transthoracic echocardiogram four-chamber view (diastolic image on (a), systolic image on (b)) demonstrating classic pattern of apical akinesis and hypercontractility of midbasal segments of the LV (arrows on (b) emphasizing this specific pattern of wall motion abnormalities seen in Takotsubo's).

Transthoracic echocardiogram (TTE) (Figure 3) was performed and revealed a preserved ejection fraction, left ventricular apical hypokinesis, a mildly enlarged right ventricle with normal systolic function, and no significant valvular pathologies. Based on these findings, the patient was diagnosed with TCM. The patient was observed for 36 hours with no recurrence of chest pain. The patient was initiated on goal-directed medical therapy and discharged from the hospital in stable condition. There were plans for close outpatient cardiology follow-up and repeat TTE in 2 months but was subsequently lost to follow-up.

## 7. Discussion

TCM is characterized by left ventricular systolic dysfunction, in the absence of obstructive coronary artery disease. Currently, about two percent of all patients undergoing emergency coronary angiography for suspected ACS have TCM [1]. About 90% of patients with TCM are postmenopausal women [1]. However, TCM in pregnancy or peripartum state is extremely rare. In general, acute cardiac complications during pregnancy or in the peripartum periods are difficult to differentiate at times. The two most common are ischemic heart disease and peripartum cardiomyopathy [3]. Peripartum cardiomyopathy occurs in 1 per 3,000 births and typically presents in the last month of pregnancy or in the first five months postpartum [1, 3]. As opposed to TCM, which presents more similarly to ACS, patients with peripartum cardiomyopathy present with signs and symptoms of congestive heart failure. TCM also differs in that it can develop shortly after delivery as opposed to peripartum cardiomyopathy that can take months to develop or manifest clinically. TCM can also present in the postpartum period with coexisting conditions associated with a heightened sympathetic response. Acute intracranial disease processes and injuries have been shown to be possible triggers for TCM. For example, a clinical case demonstrated a patient who initially presented with unrelenting headaches in the postpartum period with reversible cerebral vasoconstriction who developed chest pain and was diagnosed with TCM [4]. Furthermore, another rare trigger for postpartum TCM that has been cited is pheochromocytoma. Peripartum pheochromocytoma is difficult to diagnose as the symptoms can often mimic preeclampsia [5]. Establishing the trigger for TCM postpartum is vital so that management can be altered to focus on the underlying etiology.

Peripartum cardiomyopathy is more widely understood, and risk factors include older age, black race, preeclampsia, multiparity, and twin gestations [3]. Our patient had many of the risk factors usually associated with peripartum cardiomyopathy, including multiparity, twin gestations, and a previous pregnancy complicated by preeclampsia. Differentiating between the two syndromes is very important as they require different management and follow-up. For example, beta-adrenergic inotropic agents can be used in peripartum cardiomyopathy, while it is contraindicated in TCM [6]. Additionally, left ventricular function tends to recover fully and recovers more rapidly in TCM as compared to peripartum cardiomyopathy [5, 6]. See Table 1 for key differences between possible cardiac complications during pregnancy and the peripartum period.

The nomenclature and diagnostic criteria behind TCM continue to evolve. Initially, TCM was defined by Mayo's diagnostic criteria which was established in 2004 [7]. This includes hypokinesis of the left ventricular mid segment, with or without apical involvement, absence of coronary disease on angiography, new EKG or troponin abnormalities, and the absence of myocarditis or pheochromocytoma [7, 8]. Our patient fulfilled all these criteria which established the diagnosis. More recent research continues to further expand the diagnostic basis behind TCM and have separated it into “primary” and “secondary.” In primary TCM, the initial stressor may not be clear, and the patient's cardiac symptoms are the main reason for seeking medical care. Secondary TCM requires the patient to be already hospitalized, whether for surgical, psychiatric, or obstetric reasons [9]. As in our patient, the inciting stressful trigger is presumed to be her pregnancy, which imposes both emotional and physical stressors. This is a valuable distinction as TCM should be included in the differential diagnosis of other pregnancy-related complications, such as peripartum cardiomyopathy, spontaneous coronary artery dissection (SCAD), and eclampsia.

## 8. Conclusions

In conclusion, although it is rare, TCM can present during pregnancy. Recognition of this condition and early diagnosis are important as it can rarely lead to cardiac rupture and death. It is also vital to distinguish this condition from the widely understood and more common cardiac complication seen in pregnancy—peripartum cardiomyopathy. Once diagnosed, management of hospitalized patients with TCM consists of close monitoring of hemodynamics inpatient for 1-2 days, as in our patient. All patients should have close cardiology follow-up with serial echocardiograms to monitor for improvement in LV function. Supportive care with goal-directed medical therapies for LV dysfunction and heart failure if present should be instituted in patients with TCM. Most patients will recover normal cardiac function within 4-8 weeks [7]. Unfortunately, our patient was lost to clinical follow-up and a repeat echocardiography was not done. However, although recurrence in premenopausal women is very rare, all patients should be counseled of the potential risks in further pregnancies.

## Figures and Tables

**Table 1:** Cardiac complications during pregnancy—distinctive features.

Condition	Timing	Cardiac biomarkers	LV dysfunction	LHC findings	Risk factors	Course recurrence/prognosis	Treatment
TKSCM^1^	Antepartum, intrapartum, and/or immediate postpartum period	+	Present	Normal	Psychosocial stressors, comorbid psychological disorders	Recovery in 4-8 weeks; rare recurrence	Supportive care or guideline-directed medical therapy (GDMT) for heart failure with reduced ejection fraction
PPCM^3^	One month prepartum-5 months postpartum	+/−	Present	Normal	Advanced maternal age, preeclampsia, multiparity, twin gestations, black race	Recovery in 3-6 months; high recurrence rate	GDMT for heart failure with reduced EF
SCAD^10^	Third trimester-early postpartum	+	Absent	Radiolucent lumens	Black race, advanced maternal age, chronic hypertension, and preeclampsia	Variable prognosis; may recur	May require PCI, medical therapy, cardiac rehabilitation
Pre/eclampsia^11^	Third trimester-decades later	+/−	Present or absent	Normal or coronary artery disease (increased risk later in life)	Advanced maternal age, black race, obesity, smoking, hypertension	Leads to increased risk for CAD, heart failure, stroke	Preventative measures, screening, and treatment of comorbidities

## Abbreviations

TCM: Takotsubo cardiomyopathy
SCAD: Spontaneous coronary artery dissection
TnT: Troponin T
LHC: Left heart catheterization
STEMI: ST-elevated myocardial infarction
ACS: Acute coronary syndrome.

## Additional Points

*Learning Objectives*. (i) To have a high index of suspicion for TCM in the peripartum period in women presenting with symptoms resembling an acute coronary syndrome. (ii) To recognize key differences (risk factors, imaging findings, and prognosis) in cardiac complications in pregnancy and peripartum period.
